# Periodontal Status and Gingival Crevicular Fluid *Fusobacterium nucleatum* and Cathepsin K Assessment in Patients with Gastric Cancer

**DOI:** 10.3390/jcm14196768

**Published:** 2025-09-25

**Authors:** Flavia Mirela Nicolae, Mihai Cucu, Sandu Râmboiu, Virgil Mihail Boldeanu, Adina Turcu-Stiolica, Valeriu Marin Șurlin, Dorin Nicolae Gheorghe, Dora Maria Popescu, Victor Dan Eugen Strâmbu, Radu Petre, Andreea Cristiana Didilescu, Petra Șurlin

**Affiliations:** 1 Department of Periodontology, Faculty of Dental Medicine, Research Center of Periodontal-Systemic Interactions, University of Medicine and Pharmacy of Craiova, 200349 Craiova, Romania; 2 Department of Genetics, University of Medicine and Pharmacy of Craiova, 200349 Craiova, Romania; 3 Department 1st of Surgery, Clinical County Emergency Hospital of Craiova, University of Medicine and Pharmacy of Craiova, 200349 Craiova, Romania; 4 Department of Immunology, Faculty of Medicine, University of Medicine and Pharmacy of Craiova, 200349 Craiova, Romania; 5 Department of Pharmacoeconomics and Statistical Analysis, Faculty of Pharmacy, University of Medicine and Pharmacy of Craiova, 200349 Craiova, Romania; adina.turcu@gmail.com; 6 Department of Surgery, University of Medicine and Pharmacy “Carol Davila”, 020021 Bucharest, Romania; 7 Department of Embryology and Microbiology, Faculty of Dentistry, “Carol Davila” University of Medicine and Pharmacy, 020021 Bucharest, Romania; andreea.didilescu@umfcd.ro

**Keywords:** *Fusobacterium nucleatum*, cathepsin K, periodontal disease, gastric cancer

## Abstract

**Background/Objectives**: Periodontal disease, the most widespread chronic inflammatory non-communicable disease, is caused by the host-mediated inflammatory and immune responses to the bacterial biofilm. *Fusobacterium nucleatum* contributes to its progression and is associated with gastrointestinal cancers, including gastric cancer (GC), by promoting inflammation and immune evasion. Additionally, cathepsin K (CTSK) enhances tumor invasiveness and metastasis, playing a crucial role in GC progression. **Aim**: The present study was carried out to evaluate the possible association between the amount of *F. nucleatum* from gingival crevicular fluid and the periodontal status with the characteristics of GC. **Methods**: This cross-sectional study included 48 GC patients with periodontal changes, presenting to the Surgery Department in Craiova, Romania, from March 2023 to February 2024. Clinical assessments, where the number of teeth present, probing depth (PD), and bleeding on probing (BOP) were recorded, gingival crevicular fluid sampling, qPCR for *Fusobacterium nucleatum*, and ELISA for cathepsin K were performed. Histological analysis evaluated tumor characteristics, where tumor dimension (TD) and differentiation grade (DG) were recorded, and statistical analysis was conducted using R software. **Results**: Smokers presented higher PD and *F. nucleatum* levels than non-smokers. Gender had no impact on PD, BOP, CTSK, *F. nucleatum*, or TD. PD correlated with *F. nucleatum*, TD, and CTSK. *F. nucleatum* strongly correlated with CTSK and TD, and CTSK was strongly correlated with TD. **Conclusions**: These results suggest an association between *F. nucleatum*, periodontal parameters, and characteristics of GC but future studies are needed.

## 1. Introduction

Periodontal disease is the most widespread chronic inflammatory non-communicable disease that affects people globally, ranking sixth in terms of frequency [1,2]. In the majority of cases, periodontal illnesses may be prevented and treated, but if treatment fails to take place, the tooth-supporting tissues gradually degenerate and eventually lead to tooth loss [2]. Its primary features include the loss of periodontal tissue support, which shows up as clinical attachment loss, gingival bleeding, periodontal pocketing, and alveolar bone loss detected by radiography [3]. The primary etiological factor for the development and progression of periodontal disease is the host-mediated inflammatory and immune responses to the accumulation of an organized aggregation of bacteria inside a complex intercellular matrix, known as the tooth plaque biofilm, to which are added a multitude of risk factors such as gender, smoking, diabetes mellitus, and many other local predisposing factors [2,4]. A new microbial model of periodontal pathogenesis has been developed as a result of our significantly improved understanding of the dynamic interactions between the various microbial and host factors. This model suggests that the pathogenic process that causes the destruction of periodontal tissue is not associated with a limited number of perio-pathogenic species, but rather results from the synergistic action of dysbiotic microbial communities [5]. The development of periodontal disease is closely linked to *Fusobacterium nucleatum*, a keystone perio-pathogen that binds to the majority of oral bacteria via numerous adhesins and also acts either as a metabolic cornerstone or structural support [6,7].

Based on current knowledge, *F. nucleatum* may play a role in the pathophysiology of numerous human diseases, including malignancies such as gastrointestinal tumors or breast cancer [7]. Additionally, it has been discovered to be more common outside of the oral environment [8]. Because it alters the gastrointestinal antitumor immune system (i.e., tumor-infiltrating T cells) and increases the expression of pro-inflammatory/oncogenic genes, certain strains of oral pathobionts (e.g., *Porphyromonas gingivalis* and *F. nucleatum*) may translocate through the hematogenous and enteral routes and are linked to esophageal, gastric, pancreatic, and colorectal carcinogenesis [9]. More recent studies have shown that it can also be found in a variety of gastrointestinal and extra-gastrointestinal tumors, which have been repeatedly correlated with a poor prognosis [10,11,12].

Gastric cancer (GC) is the fourth most common cause of cancer-related mortality globally and one of the most prevalent cancers. Genetic and environmental variables, such as smoking, alcohol consumption, or male gender have an impact on the onset and course of GC, which is an illness with multiple components [12,13]. Apart from *Helicobacter pylori*, a well-known risk factor for GC, the entire complex gastrointestinal tract microbiota might lead to additional carcinogenic consequences [7,12]. GC patients who are co-infected with *F. nucleatum* and *H. pylori* have a poorer prognosis, according to a cohort study, suggesting that these two bacteria work in conjunction to promote GC invasiveness [14]. Interestingly, the influence of *F. nucleatum* seems to vary depending on the type of GC; patients who test positive for *F. nucleatum* have a worse prognosis when they suffer from Lauren diffuse GC, while intestinal GC patients did not show any significant difference [15]. A Taiwanese investigation found that *F. nucleatum* colonization might change actin filament dynamics and increase GC cells’ invasiveness and mobility [16]. According to research, *F. nucleatum* promotes a number of inflammatory and carcinogenic characteristics of cells by using FadA to bind to E-cadherin and activate β-catenin signaling [17].

As a member of the C1A family’s cathepsin L-like cluster, cathepsin K (CTSK) is often investigated as a functional molecule associated with osteoclasts, which are essential for bone remodeling. In many malignancies, increased CTSK activity is closely linked to the onset and recurrence of metastasis, such as tumor invasion, progression, and bone and lymph node metastasis [18,19,20,21,22]. Another condition in which CTSK expression becomes elevated is GC, where coronin 3 strongly correlates with GC lymph node metastases and a poor prognosis through the stimulation of MMP-9 and CTSK expression [21,23]. CTSK has been shown to be a gene involved in TMN staging, survival prognosis, and, potentially, the tumor microenvironment (TME) component in GC patients [21].

Through enhancing GC cells’ invasive potential, participating in tumor-associated epithelial–mesenchymal transition (EMT), and promoting the development of an immunosuppressive TME, CTSK may aid in the initiation and progression of GC. CTSK, subsequently, becomes essential for tumor immune evasion and thus a viable option for suppressing GC cell invasion [22]. Moreover, CTSK could serve as a biomarker for the early detection of GC [21].

*Aim:* The present study was carried out to evaluate the possible association between the amount of *F. nucleatum* from gingival crevicular fluid with the characteristics of GC, and the periodontal status, additionally assessing CTSK.

## 2. Materials and Methods

### 2.1. Study Design

The current cross-sectional study was created in compliance with STROBE guidelines [24].

### 2.2. Setting

An informed consent form was provided by each patient included in the study, which was conducted in total compliance with the World Medical Association Declaration of Helsinki.

The University of Medicine and Pharmacy Craiova and Clinical County Hospital of Emergency in Craiova Research Ethics Commissions granted ethics approval for this study (Research Ethics Commissions of the University of Medicine and Pharmacy Craiova No. 85/27.10.2022 and Clinical County Hospital of Emergency in Craiova No. 1454/10.10.2022).

The study was conducted from March 2023 to February 2024. The patients were recruited from the Emergency Clinical County Hospital in Craiova, Romania—the 1st Department of Surgery.

### 2.3. Participants

The study participants were patients with GC who underwent surgery in the 1st Department of Surgery of the Emergency Clinical County Hospital of Craiova, and exhibited periodontal changes.

Individuals with GC who could comprehend simple instructions and had more than 10 teeth and at least bleeding on probing (BOP) > 10% [3] as an expression of periodontal changes, were eligible to participate in this study. Patients who were unconscious, unable to sign the informed consent form, or unable to undergo an oral examination were excluded.

The sample of our study was represented by 48 patients with GC and periodontal changes, aged 39 to 79, with a mean age of 61.4 ± 11.6. The study sample was distributed by gender: 26 (54.2%) were males and 22 (45.8%) were females. More than half of the patients, 27 (56%), were smokers.

### 2.4. Clinical Assessments

The same well-trained dentist (F.M.N.) performed an oral clinical examination on the GC patients at the Dental Medicine Department, recording the following parameters from the periodontal chart: the number of teeth present, probing depth (PD), and BOP. With the exception of the third molars and any remaining root tips, every tooth was inspected using a UNC15 periodontal probe (Hu-Friedy, Chicago, IL, USA) at six distinct sites for each tooth (mesio-vestibular, centro-vestibular, disto-vestibular, mesio-lingual, centro-lingual, and disto-lingual), with regard to the immediate full millimeter.

The patient’s periodontal chart was used to record the number of teeth that were present, excluding any remaining tooth roots. For each tooth that was present, PD was measured at six different locations and noted in the patient’s periodontal chart. These values were added up and divided by the number of locations analyzed, to determine the PD for each subject. Millimeters were used to express PD. The same six sites used for PD were used for the BOP assessment, and each site’s presence or lack of BOP was noted. Sites with BOP were added up, divided by the total number of sites analyzed, and then multiplied by 100. At the end, the percentage of bleeding areas was noted. The periodontal diagnosis for each patient was established based on all necessary parameters but not used in the statistical analysis to avoid splitting them into groups that were too small.

### 2.5. Gingival Crevicular Fluid Sampling

The gingival crevicular fluid (GCF) was obtained from the tooth with the deepest probing depth or the tooth with the most severe BOP following the periodontal clinical assessment. Cotton rolls were used to isolate each tooth, which was afterwards air-dried. Another cotton roll was used to remove the supragingival plaque. Two samples, one for each compound to assess, were taken at one-minute intervals. The intra-crevicular approach was used to obtain two GCF samples through the absorption technique and sterile paper cones of size 60 (Dentsply-De-Trey^®^, Ballaigues, Switzerland) were used, by being placed for 30 s in the gingival sulcus. The Periotron 8000 equipment (Oraflow Inc., Smithtown, NY, USA) was used to quantify the GCF volume after the cones were removed from the periodontal pocket/gingival sulcus and visually examined for the absence of blood stains. Following that, one paper strip was inserted into a polyethylene microtube containing 50 µL of phosphate-buffered saline (PBS). The second paper was put in 2 mL cryotubes containing 500 µL of RNA Save^®^ solution (Biological Industries, Haemek, Israel). The samples were kept in storage at −80 °C until they were needed for DNA extraction and immunological assessment.

### 2.6. PCR Assessment

Bacterial DNA extraction and quantitative polymerase chain reaction (qPCR) were carried out as follows. Genomic DNA was extracted from crevicular fluid samples using the PureLink^®^ Genomic DNA Mini Kit (Invitrogen, Carlsbad, CA, USA), following the manufacturer’s guidelines. The concentration of DNA was quantified using the NanoDrop-2000 Micro Volume UV Spectrophotometer (NanoDrop Corporation, Waltham, MA, USA). For the detection of *Fusobacterium nucleatum*, the sequences of the primers employed were as follows: the forward primer was 5′-AGAGTTTGATCCTGGCTCAG-3′, and the reverse primer was 5′-GTCATCGTGCACACAGAATTGCTG-3′.

To construct the standard curve and serve as a positive control, the recombinant plasmid pUC57 was utilized. This plasmid contains the DNA sequence of interest flanked by complementary sequences to the primers, facilitating their attachment. The synthetic plasmid was procured from ThermoScientific LSG (Waltham, MA, USA). Pure water was employed as a negative control, serving as a substitute for the DNA isolated from the test sample.

Each qPCR reaction utilized 80 ng of DNA in a total volume of 10 µL. The amplification and detection of DNA were performed using a ViiA7 real-time PCR system (Thermo Fisher Scientific, Waltham, MA, USA) under the following conditions: an initial denaturation step at 95 °C for 10 min, followed by 40 cycles consisting of 15 s at 95 °C and 1 min at 58 °C. Negative controls were included in the reaction and consisted of diethyl pyrocarbonate (DEPC)-treated water samples.

### 2.7. Immunological Assessment

Using ELISA test kits specifically designed for the detection and quantification of cathepsin K (sensitivity: 0.1 ng/mL; intra-assay: CV (%) = 5.12; inter-assay: CV (%) = 6.42; and recovery: range (%) = 86–97; Elabscience Houston, TX, USA), the immunological analysis of GCF samples was carried out at the University of Medicine and Pharmacy’s Immunology Laboratory in Craiova, Romania. An optical analyzer was used to obtain readings at 450 nm, and these were corrected at 540 nm.

### 2.8. Histological Analysis of the Gastric Tissue

Using the standard histopathological technique, the same pathologist analyzed the GC tissue samples from gastrectomies in the Pathology Laboratory of the Clinical County Hospital of Emergency of Craiova. The tissue sample that had been collected was fixed with a 10% formalin solution. Following the steps of dehydration, clarity, paraffining, actual inclusion, sectioning, and staining, the pieces that were collected during the macroscopic orientation of the piece were processed using the traditional histological procedure of inclusion in paraffin. Hematoxylin–eosin staining was employed for the basic histopathological analysis.

On the histopathological examination, tumor dimension (TD) and differentiation grade (DG) were recorded. The DG was noted as follows for all gastric samples: G1—well-differentiated, G2—moderately differentiated, and G3—poorly differentiated in comparison to healthy adjacent tissues. Regarding TD, the neoplastic tissue’s maximum diameter was measured in millimetres.

### 2.9. Statistical Analysis

We conducted a statistical analysis in R (R Core Team 2022, version 4.2.2 for Windows), using the *ggplot2* package for data analysis and visualization. Descriptive statistics were applied, with continuous variables reported as mean ± standard deviation (SD) or median (interquartile range), and categorical variables presented as counts and percentages. The normality of continuous variables was evaluated using the Shapiro–Wilk test, with a *p*-value ≤ 0.05 indicating a rejection of the null hypothesis of normality. For group comparisons between male and female patients or between smokers and non-smokers, the nonparametric Mann–Whitney U test was applied. To account for multiple testing and reduce the risk of a type I error, *p*-values were adjusted using the Benjamini–Hochberg false discovery rate (BH FDR) procedure. We also reported the Benjamini–Hochberg adjusted p-value as a q-value and 0.1 was considered statistically significant. Correlations between periodontal parameters, tumor characteristics, and the relative abundance of *F. nucleatum* and cathepsin K were evaluated using Spearman’s rank correlation coefficients. Results were visualized in a heatmap correlation matrix, where green represented positive correlations (Spearman’s ρ approaching 1) and orange indicated negative correlations (Spearman’s ρ approaching −1). A two-sided *p* < 0.05 was considered statistically significant for all analyses. Post hoc power analysis was conducted using G*Power (version 3.1.9.7) and the conventional 80% threshold was used.

## 3. Results

### 3.1. Periodontal Parameters

The periodontal parameters are described in Table 1.

### 3.2. F. nucleatum and Cathepsin K

GCF levels of *F. nucleatum* and CTSK are described in Table 2.

### 3.3. Tumor Characteristics

The differentiation grades were almost equally represented: G1 in 16 patients (33.3%), G2 in 15 patients (31.2%), and G3 in 17 patients (35.4%).

Regarding the tumor dimension, the mean value ± SD was 91.4 ± 26.7, ranging from 50 to 135 mm.

### 3.4. Comparisons Between Smokers and Non-Smokers

**Figure 1:**
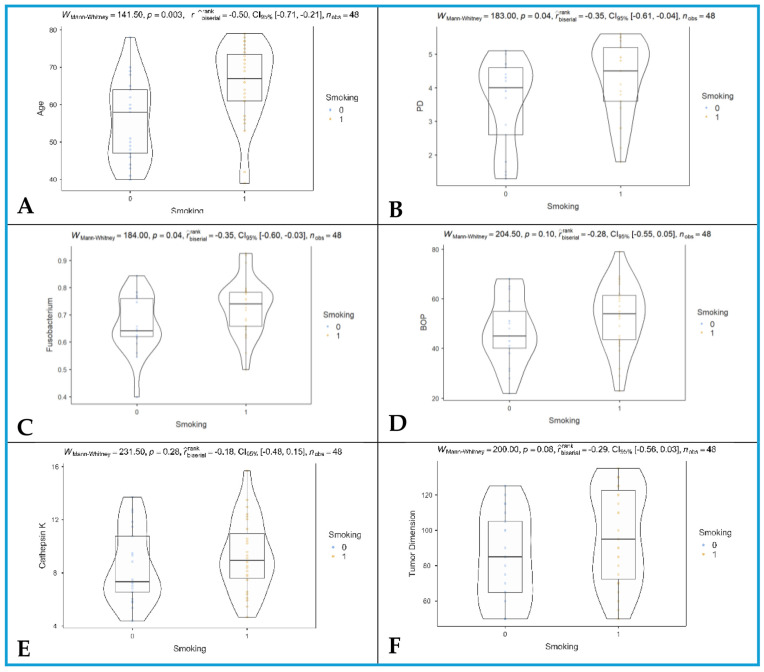
Comparisons between smokers (coded with 1) and non-smokers (coding with 0). (**A**) Age; (**B**) PD, probing depth; (**C**) *Fusobacterium*, *Fusobacterium nucleatum*; (**D**) BOP, bleeding on probing; (**E**) cathepsin K; and (**F**) tumor dimension.

In our study, older patients were more likely to smoke compared to younger patients (effect size, rank-biserial r = −0.50, 95% CI = [−0.71, −0.21], and q = 0.0006; Figure 1A). Smoking patients exhibited higher PD compared to non-smoking patients (effect size, rank-biserial r = −0.35, 95% CI = [−0.61, −0.04], and q = 0.08; Figure 1B). Higher levels of *F. nucleatum* were observed in smoking patients (effect size, rank-biserial r = −0.35, 95% CI = [−0.60, −0.03], and q = 0.08; Figure 1C).

BOP values were comparable between smoking and non-smoking patients (effect size, rank-biserial r = −0.28, 95% CI = [−0.55, 0.05], *p* = 0.10, and q = 0.12; Figure 1D). CTSK levels were comparable between smoking and non-smoking patients (effect size, rank-biserial r = −0.18, 95% CI = [−0.48, 0.15], and q = 0.28; Figure 1E). TDs were similar between smoking and non-smoking patients (effect size, rank-biserial r = −0.29, 95% CI = [−0.56, 0.03], and q = 0.12; Figure 1F).

After BH FDR correction, age, PD, and *F. nucleatum* comparisons remained significant, whereas TD, BOP, and CTSK comparisons were not significant.

A post hoc power analysis was conducted for the Wilcoxon signed-rank test and the achieved power ranged between 45% and 84% indicating a moderate to high probability of correctly rejecting the null hypothesis if the true effect exists.

### 3.5. Comparisons Between Males and Females

**Figure 2:**
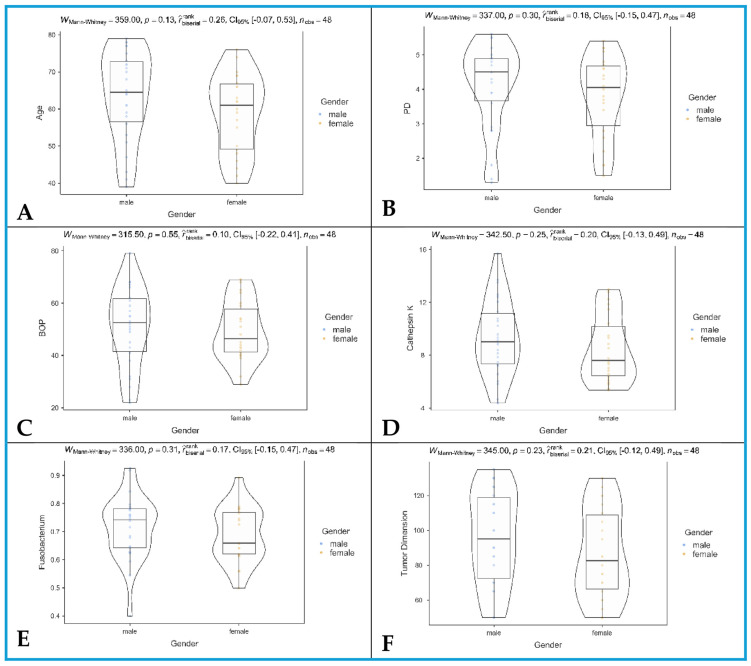
Comparisons between males and females. (**A**) Age; (**B**) PD, probing depth; (**C**) BOP, bleeding on probing; (**D**) cathepsin K; (**E**) *Fusobacterium*, *Fusobacterium nucleatum*; and (**F**) tumor dimension.

In our study, no differences regarding age were found between males and females (effect size, rank-biserial r = 0.26, 95% CI = [−0.07, 0.53], and q = 372; Figure 2A). Regarding higher PD, no differences were found between male and female patients (effect size, rank-biserial r = 0.18, 95% CI = [−0.15, 0.47], and q = 0.372; Figure 2B). BOP values were comparable between male and female patients (effect size, rank-biserial r = 0.10, 95% CI = [−0.22, 0.41], and q = 0.55; Figure 2C). CTSK levels were comparable between male and female patients (effect size, rank-biserial r = 0.20, 95% CI = [−0.13, 0.49], and q = 372; Figure 2D). No differences in *F. nucleatum* levels were observed between male and female patients (effect size, rank-biserial r = 0.31, 95% CI = [−0.15, 0.47], and q = 0.372; Figure 2E). TDs were similar between male and female patients (effect size, rank-biserial r = 0.21, 95% CI = [−0.12, 0.49], and q = 0.372; Figure 2F).

After BH FDR correction, no comparisons remained significant.

### 3.6. Correlations Between Periodontal Parameters, Tumor Characteristics, and Amount of Fusobacterium nucleatum and Cathepsin K

**Figure 3:**
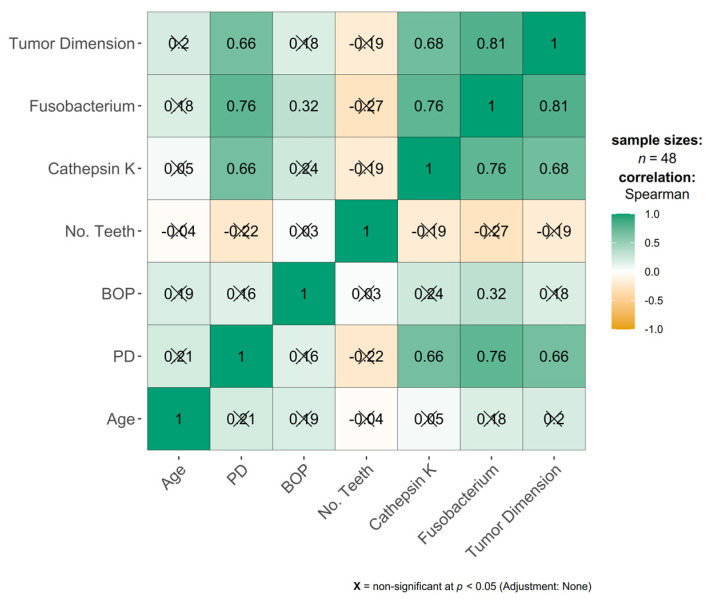
Correlation matrix. Green = strong positive correlation (rho = 1); orange = strong negative correlation (rho = −1). PD, probing depth; BOP, bleeding on probing; No. Teeth, number of teeth present; and *Fusobacterium*, *Fusobacterium nucleatum*.

Our results showed that PD was strongly correlated with *F. nucleatum*, with high statistical significance (ρ = 0.76, *p* < 0.001), and moderately correlated with TD (ρ = 0.66, *p* < 0.001) and CTSK (ρ = 0.66, *p* < 0.001), with high statistical significance. PD was weakly correlated with smoking and the association was borderline significant (ρ = 0.31, *p* = 0.035), as shown in Figure 3.

BOP was weakly but statistically significantly correlated with *F. nucleatum* (ρ = 0.32, *p* = 0.026).

*F. nucleatum* demonstrated a strong and highly significant correlation with CTSK (ρ = 0.76, *p* < 0.001) and TD (ρ = 0.81, *p* < 0.001) and a weak significant correlation with smoking (ρ = 0.30, *p* = 0.037).

CTSK was strongly and highly statistically significant correlated with TD (ρ = 0.68, *p* < 0.001).

## 4. Discussion

Following our previous pilot study [25], we showed that *F. nucleatum* was the most prevalent perio-pathogen in the gingival fluid of individuals with gastric cancer out of all of those examined. Those findings, together with the correlations found between tumor dimension and *F. nucleatum*, prompted us to design the current study to further investigate the involvement of this bacterium in gastric cancer pathology, in an attempt to find associations between it and tumor characteristics.

Smoking has been linked to periodontal disease through a number of mechanisms, such as shifts in the subgingival microbial communities, modifications of the host and inflammatory responses to potential periodontal pathogens, and a weakened tissue’s capacity to heal, which results in an imbalance of tissue homeostasis [26]. Our comparisons showed that smokers had increased probing depths and levels of *F. nucleatum*. Previous studies clearly stated that smokers have more deep pockets, greater probing depths, and more attachment loss, including gingival recession [27,28,29]. Rather than having a vasoconstrictive effect, the evidence that is nowadays available indicates that smoking has a long-term impact by affecting the periodontal tissues’ vasculature. In addition to reducing gingival redness and bleeding on probing, the reduced vasculature may also hinder the healing response by influencing revascularization [28,29,30]. In our study, the smoking status was positively correlated with probing depths, but not with bleeding on probing.

Even though several studies found no difference between smokers, either conventional smokers or electronic cigarette smokers, and non-smokers in terms of oral microbiota, data from other studies indicated that smokers tended to have more periodontal pathogens than non-smokers without having more plaque, often enriched in *F. nucleatum* and *Bacteroidales* [29,30,31]. Our results also point towards a positive correlation between smoking and *F. nucleatum*.

Cigarette smoking is an independent risk factor for GC, especially for the gastric cardia, according to a recent meta-analysis [32]. According to estimates, tobacco use is responsible for 18% of GC cases [33]. Regarding the characteristics of the malignancies, the tumor dimensions did not differ between smoking and non-smoking patients; even though smoking is a well-known risk factor for the development of gastric cancer, the scientific literature is scarce when it comes to its smoking-induced evolution.

According to reports, smoking increases the risk of GC by 60% for males and 20% for women [34,35]. Gastric cancer is more prevalent in men, demonstrating substantial gender inequalities. Men experienced a 2.5-fold increased risk of stomach cancer over the past ten years compared to women, with the largest difference seen in those aged 60–64. Gender discrepancy may be shaped by differences in lifestyle variables such food habits and tobacco use, as well as hormonal disparities between men and women, potentially because of the protective benefits of estrogen in women, which may decrease after menopause [36,37,38]. Regarding gender, our results showed no differences for tumor dimensions or differentiation grade between males and females.

Men have a greater possibility than women to develop periodontal diseases due a variety of biological and gender-related variables, such as immune system components, hormone variations, worse oral hygiene habits, and increased tobacco use [39,40,41]. The immune response is influenced by sex chromosomes, and X-linked genes modulate the immunological response. Immunity is also impacted by sex hormones, such as progesterone, testosterone, and estrogens. According to numerous articles, estrogen generally boosts the immunological response, whereas testosterone generally decreases it [41,42,43,44]. Regarding gender, our results showed no differences between males and females, for the periodontal parameters. These variations in immunological function also impact the oral microbiota. According to Ioannidou et al., Sultan et al., and other authors, some differences in the prevalence of periodontal disease between men and women are probably due to sex-related differences in microbial populations and immunological systems [41,42,45,46,47]. We found no differences between males and females regarding the levels of *F. nucleatum* and CTSK.

The detection of CTSK in the GCF of individuals with severe, moderate, and mild periodontitis was initially reported by Mogi and Otogoto. It was interesting when researchers discovered that as the severity of periodontitis increased, the concentration of CTSK dropped [48]. Additionally, Yamalik et al. discovered that the activity of CTSK in the GCF of patients with periodontitis was substantially higher than that of normal individuals and patients with gingivitis, in addition to the change in the quantity of GCF CTSK in patients with periodontitis [49,50]. Our results also showed a strong, statistically significant correlation between probing depths and GCF levels of CTSK, even though a periodontitis diagnosis was not established, as it was not our aim.

Smokers with chronic periodontitis had considerably higher levels of CTSK than nonsmokers, indicating that smoking has a positive impact on CTSK, which may contribute to smokers’ greater susceptibility to osteoclastic bone deterioration [51]. In our study, we found comparable levels of CTSK in smokers and non-smokers. Regarding the periodontal parameters, bleeding on probing was similar for both groups, but the probing depths were statistically higher in smokers.

In addition to confirming that GCF CTSK levels rose in cases of severe periodontitis, Garg et al. discovered that CTSK levels dropped by 60% following non-surgical periodontitis therapy [52]. Administering AAV-sh-CTSK can successfully prevent alveolar bone loss and injury to periodontal tissue, making AAV-mediated local CTSK silencing an important therapeutic approach for the effective treatment of periodontal disease [53].

Numerous types of periodontal illnesses, including the mild, reversible form of gingivitis and the more severe, irreversible forms, such as chronic, localized, and generalized aggressive periodontitis, are linked to *F. nucleatum* [54]. As inflammation progresses, pockets deepen, and illness severity increases, so does the amount of *F. nucleatum* [55,56,57]. In our study, GCF levels of *F. nucleatum* were positively and statistically correlated with periodontal parameters, like bleeding on probing and probing depths.

Epidemiologic surveys have additionally linked periodontitis and exposure to oral pathobionts to a higher risk of cancer incidence and death [58,59,60]. According to one study, the incidence of gastric cancer was positively correlated with self-reported periodontitis [61]. In a comparable manner, a large-scale study conducted in South Korea with 3920 cases among over 700,000 participants discovered a modest yet significant connection between stomach cancer and clinically measured periodontitis [62]. Our results showed that the probing depths, an important periodontal parameter, were strongly and significantly correlated with tumor dimensions.

*F. nucleatum* expression has been found in GC patients in recent research [63,64]. According to the findings of Hsieh et al.’s study, *H. Pylori* was underrepresented in the stomach microbiota of GC patients, but *Fusobacterium* and *Clostridium*—including *F. nucleatum*—were extremely abundant [65]. Furthermore, it was shown that patients with diffuse Lauren-type GC who had *F. nucleatum* identified had a substantially lower overall survival rate [15]. However, the particular approach by which *F. nucleatum* influences GC has not yet been investigated. Various mechanisms have been implicated, such as the propagation of carcinogenic oral pathobionts and the development of a persistent systemic inflammatory state. Periodontitis may, in fact, support low-grade systemic inflammation and phenotypic alterations in mononuclear cells, which can result in the production of cytokines and free radicals as well as the breakdown of extracellular matrix—all of which are processes that contribute to the development of cancer and its spread. Furthermore, non-indigenous bacterial colonization of many microenvironments may result from the temporary hematogenous spill out or micro-aspiration/swallowing of periodontal bacteria and their virulence components. These occurrences might then affect the molecular hallmarks of cancer by repopulating the tumor-associated microbiota [9].

The fact that *F. nucleatum* could infect and penetrate GC cells was noted by Xin et al., indicating that the bacteria could reside in the stomach in this manner and contribute to the development of GC [16]. Our results showed that GCF levels of *F. nucleatum* were positively associated with the dimensions of the gastric tumors.

According to one study, CTSK mediates the relationship between gut microbiota imbalance and colorectal cancer metastases. Both in vitro and in vivo, CTSK plays a role in the aggressive phenotype of colorectal cancer cells. Furthermore, they demonstrated a positive feedback loop mediated by CTSK between CRC cells and tumor-associated macrophages during metastasis, establishing CTSK as a potential therapeutic target and new prognostic biomarker for CRC [66,67]. We found statistically significant correlations between the GCF levels of *F. nucleatum* and CTSK in GC patients.

A recent study reported a notable increase in CTSK expression not only in tumor tissues compared to adjacent healthy tissues, but in high-stage tumors, when compared with low-stage tumors. This finding indicates a possible involvement of CTSK in facilitating oncogenesis in human gastric tissues [22]. The prognosis for patients with high CTSK expression was substantially poorer than that of those with low expression [21]. Research indicates that the metastasis of gastric cancer related to coronin 3, a highly conserved regulator of the actin cytoskeleton, is at least partially facilitated by MMP-9, TIMP-2, and cathepsin K [23].

Through increasing GC cells’ ability to invade and inducing an inflammatory response around tumor cells, an increased expression of CTSK may have an effect on the progression of GC. The results confirmed that CTSK over-expression does, in fact, promote GC invasion and growth [22]. Even though we did not determine CTSK in tumorous tissues, their GCF levels were significantly correlated with the tumor dimension. In vitro and in vivo, Ren et al. have demonstrated explicitly that coronin 3 is highly expressed in metastatic gastric cancer tissues, contributing to the invasion and metastasis process. Additionally, they have shown that coronin 3 can facilitate gastric cancer metastasis, at least in part, by up-regulating the expression of MMP9 and CTSK and down-regulating that of TIMP2 [23].

Angiogenesis, EMT, inflammatory response, TNFα signaling via NFκB, and IL6-JAK-STAT3 signaling are hallmark gene sets linked to carcinogenesis and cancer-related inflammation, and they were activated in GC patients with elevated CTSK expression. Furthermore, the in vitro data clearly show that an over-expression of CTSK promotes GC cells’ invasive and proliferative potentials while having little to no impact on their capacity to migrate [22].

The most prevalent endosomal/lysosomal cysteine proteases, cathepsin B and L, have been shown by Farinati et al. to be elevated in the latter stages preceding gastric cancer, such as atrophic gastritis and epithelial dysplasias [68]. In comparison to non-neoplastic mucosa, gastric cancer also showed a 3–12 fold increase in cathepsin X [69]. Additionally, the prognosis and stage of the tumor are correlated with blood levels of cathepsin B [70]. In vitro, increased invasiveness was directly linked to up-regulated cathepsin X. The same study shows that cathepsin X contributes to both the tumorigenesis of gastric cancer and the chronic inflammation of the gastric mucosa, despite the fact that there was no association between cathepsin X expression and TNM stage [69].

Patients with various types of cancer who are at an increased risk of bone metastases may also benefit from CTSK as a possible therapeutic target [22,71,72]. As demonstrated by the data, CTSK is a potential predictive target associated with the TME of gastric cancer, and its expression not only predicts the prognosis of patients but also offers novel approaches for tumor immunotherapy [21]. By reducing the over-expression of CTSK, a recently discovered targeted K21-based drug delivery system can decrease oral squamous cell carcinoma cells’ migration and adhesion [73].

Data from a cross-sectional study showed that the increased levels of *F. nucleatum* in patients’ saliva compared to controls represents a promising path for facilitating the development of a novel early diagnosis model for gastric cancer, a condition where early detection is hampered by the invasiveness of gastroscopy and the low specificity and sensitivity of conventional serum tumor markers [12,64]. Furthermore, several markers, including *F. nucleatum* DNA and HOTTIP, have been found to be elevated in GC patients’ blood and saliva samples [16,64,74]. This might offer a non-invasive GC detection technique.

The small number of patients who could be included in the study is a limitation of this research. The post hoc power for the observed effects ranged between 5 and 84%, necessitating future validation of these findings on larger cohorts, which would also allow the use of more groups’ characteristics in the statistical analysis.

## 5. Conclusions

These results suggest an association between *F. nucleatum*, *CTSK*, periodontal parameters, and the dimension of gastric tumors but future studies which investigate the correlation between the periodontal microbiome, periodontal changes, and tumorous gastric tissue are necessary.

## Figures and Tables

**Table 1:** Clinical periodontal characteristics of the study participants.

	Mean ± SD	Median (IQR)	Range
Number of teeth present	16.8 ± 4.76	16 (13–21)	10–27
BOP (%)	50.1 ± 13.5	51 (41–60.3)	22–79
PD (mm)	3.97 ± 1.23	4.3 (3.27–4.9)	1.3–5.6

SD, standard deviation; IQR, interquartile range; BOP, bleeding on probing; PD, probing depth.

**Table 2:** *Fusobacterium nucleatum* and cathepsin K levels in gingival crevicular fluid.

	Mean ± SD	Median (IQR)	Range
*F. nucleatum (relative abundance)*	0.7 ± 0.11	0.72 (0.63–0.78)	0.4–0.93
CTSK (ng/mL)	8.92 ± 2.72	8.66 (6.77–10.9)	4.4–15.7

SD, standard deviation; IQR, interquartile range; *F. nucleatum*, *Fusobacterium nucleatum*; CTSK, cathepsin K.

## Data Availability

Data used to support the findings of this study are available from the corresponding author upon request.

## Abbreviations

The following abbreviations are used in this manuscript:

GC	Gastric Cancer
CTSK	Cathepsin K
PD	Probing Depth
BOP	Bleeding on Probing
TD	Tumor Dimension
DG	Differentiation Grade
TME	Tumor Microenvironment
EMT	Epithelial–mesenchymal Transition
GCF	Gingival Crevicular Fluid
SD	Standard Deviation
